# History and Status of Selected Hosts of the *Ralstonia solanacearum* Species Complex Causing Bacterial Wilt in Brazil

**DOI:** 10.3389/fmicb.2018.01228

**Published:** 2018-06-13

**Authors:** Carlos A. Lopes, Maurício Rossato

**Affiliations:** ^1^Embrapa Hortaliças, Brasília, Brazil; ^2^Departamento de Fitopatologia, Universidade de Brasília, Brasília, Brazil

**Keywords:** bacterial wilt, banana, distribution, eucalyptus, potato, tomato, Moko disease

## Abstract

Bacterial wilt induced by the *Ralstonia solanacearum* species complex is endemic to Brazil, where it can cause variable losses in many hosts. Its economic importance, however, cannot be precisely measured due to Brazil’s continental size, subject to variable weather conditions which directly affect disease expression. The objectives of this paper were (i) to gather scattered information on historical facts; (ii) to show the current distribution of the pathogen in the country, and (iii) to comment on future trends on the importance of the disease in economically important current and potential hosts, based on the pathogen’s variability and the global climate change under way.

## Introduction

Brazil is considered a putative center of origin of *Ralstonia* *solanacearum* (phylotype II) (Wicker et al., 2012), but *R. pseudosolanacearum* (phylotype I) is also present in the country (Santiago et al., 2017). The diversity between and within these species is reflected in a broad genetic background and a wide host range, which is constantly increasing. The bacterial wilt (BW) disease that these species induce is responsible for substantial losses in important crops such as potato, tomato, and banana. Some other hosts reported in the country are unusual, apparently not found in other parts of the world, or are found in peculiar host/bacterial strain combinations, as indicated in Malavolta Júnior et al. (2008) and Morais et al. (2015). Among these, cassava (*Manihot esculenta*) (Robbs, 1954), long pepper (*Piper hispidinervum*) (Lopes et al., 1997), passion fruit *(Passiflora edulis)* (Lopes et al., 1999), cactus (*Cereus peruvianus monstruosus*) (Pereira et al., 2001), squash (*Cucurbita pepo*) (Sinigaglia et al., 2001), cariru (*Talinum triangulare*), amazon chicory (*Eryngium foetidum*) (Lopes et al., 2002), rocket (*Eruca sativa*) (Albuquerque et al., 2016), and chickpea (*Cicer arietinum*) (Salcedo et al., 2016) are found. However, important hosts, such as cassava, sweet potato, and peanut, subject to heavy losses due to BW in tropical countries in Africa and Asia, are not affected in South America. This might be explained by the absence of specific strains, especially those of biovar IV, phylotype III, not found in Brazil.

This article aims to review historical facts and the current status of the *R. solanacearum* species complex in three important groups of plants in Brazil: solanaceous crops, bananas, and eucalyptus.

## Brief History

### Solanaceous Crops

The first record of BW in Brazil is believed to date from the early 1920’s, by Parseval (1922), a German professor at the Instituto Borges de Medeiros, of the Escola de Engenharia de Porto Alegre (now Escola de Agronomia of Universidade Federal do Rio Grande do Sul). He first observed the disease when visiting tobacco fields in Santa Cruz, RS, associating it with a BW based on publications of E. F. Smith. Even though conclusive pathogenicity tests were not performed, the description of symptoms and observation of bacterial ooze from tobacco stem pieces were robust indications that Von Parseval’s diagnosis was correct. Furthermore, information from local growers at the time suggested that the disease was disseminating steadily in tobacco and in potato. Planting seed potato produced in infested fields was believed to be the main cause of BW spread.

In 1931, samples of wilted tomato and potato plants collected in the State of Minas Gerais were examined at the Instituto Ezequiel Dias, in Belo Horizonte, MG, by Dr. Octavio de Magalhães, who isolated a bacterium from the plant vessels, identified as *Phytomonas solanacearum* (Magalhães, 1932). However, this report is not reliable, since the author stated that the bacterium was anaerobic and caused soft rot on potato tubers, characteristic of pectolytic bacteria, suggesting a possible double infection of the examined tissues.

In 1934, a list of diseases diagnosed in 1931 and 1932 at the Plant Pathology Section of the Instituto Biológico, in São Paulo, SP, indicated that BW (*Bacterium solanacearum*) was one of the most important potato diseases in the state of São Paulo (Bitancourt, 1934). In 1935, BW was independently reported in Minas Gerais (Werner, 1935), in São Paulo (Gonçalves, 1935), and in Rio de Janeiro (Azevedo, 1935). Better description and illustrations of the symptoms were provided, as well as recommendations for disease control. Immersion of seed potatoes in pesticides containing mercury (banned in the 1980’s in Brazil) was a common practice at the time.

Costa and Krug (1937) clearly illustrated BW symptoms in potatoes in Corumbataí, SP and, apparently, were the first to quantify losses induced by the disease (20–30%). These authors, however, admitted that the pathogen was not precisely identified, but they strongly indicated that it was the same pathogen, *Bacterium solanacearum*, described by Azevedo (1935) in Rio de Janeiro. In addition, according to Kramer and Amaral (1944), based on symptomatology, the same disease was observed in So Paulo by Costa and Krug (1937); in Rio Grande do Sul by Costa Neto (1937); and in Paran by Deslandes (1941).

In the early 1940’s, with the expansion of the potato crop in the southern and southeastern regions, BW became a constraint on good yields, especially in the summer. This fact stimulated M. Kramer and J. Franco do Amaral, from the Instituto Biológico, SP, to intensify their studies to unequivocally establish the relationship between the pathogen and the host, predict possible losses and recommend control measures.

At the same time, José Alencar and Octavio Drummond, from the Universidade Federal de Viçosa, published a comprehensive report on host range, symptomatology and etiology of the BW disease based on samples of wilted potato plants collected in Viçosa, Ponte Nova, and Ouro Preto, municipalities of the State of Minas Gerais (Alencar and Drummond, 1944).

Two independent groups (Kramer & Franco do Amaral and Alencar & Drummond) were apparently the first to obtain pure cultures of the pathogen and to perform Koch’s postulates in potato and tomato, thus scientifically establishing the pathogenicity of *R. solanacearum* on two of the most important hosts in Brazil. In addition, by recognizing the destructive potential of the pathogen and its dissemination patterns, Kramer & Amaral recommended “zero tolerance” in seed-potato field inspections.

Outstanding contributions for BW control are attributed to Arnaldo Medeiros in the 1950’s (Medeiros, 1960), when he described anti-serum production and use to detect the pathogen, chemical control by fumigants, soil pH affecting disease, and association of actinomycetes with the pathogen for biological control purposes.

Octavio A. Drummond, during his long and productive career, was a professor and researcher who dedicated most of his time to BW in the states of Minas Gerais and Rio de Janeiro. In addition to his pioneering research on the elucidation of BW etiology, he installed and maintained a set of isolated *R. solanacearum*-infested nurseries in Itaguaí, Rio de Janeiro, where for many years he tested a series of combinations of plant species for crop rotation, aiming at disease control.

### Bananas and Their Relatives – Moko Disease

Moko disease is a peculiar manifestation of BW, caused by race 2, biovar 1, phylotype II of *Ralstonia solanacearum,* a quarantine pest that, at high temperatures (optimum 35–37°C) can infect triploid bananas, heliconias (*Heliconia* spp.) and other ornamental *Musaceae* plants (Hayward, 1994; OEPP/EPPO, 2004). It is present in many tropical and warm temperate countries where the hosts are important food staples or ornamentals.

**FIGURE 1:**
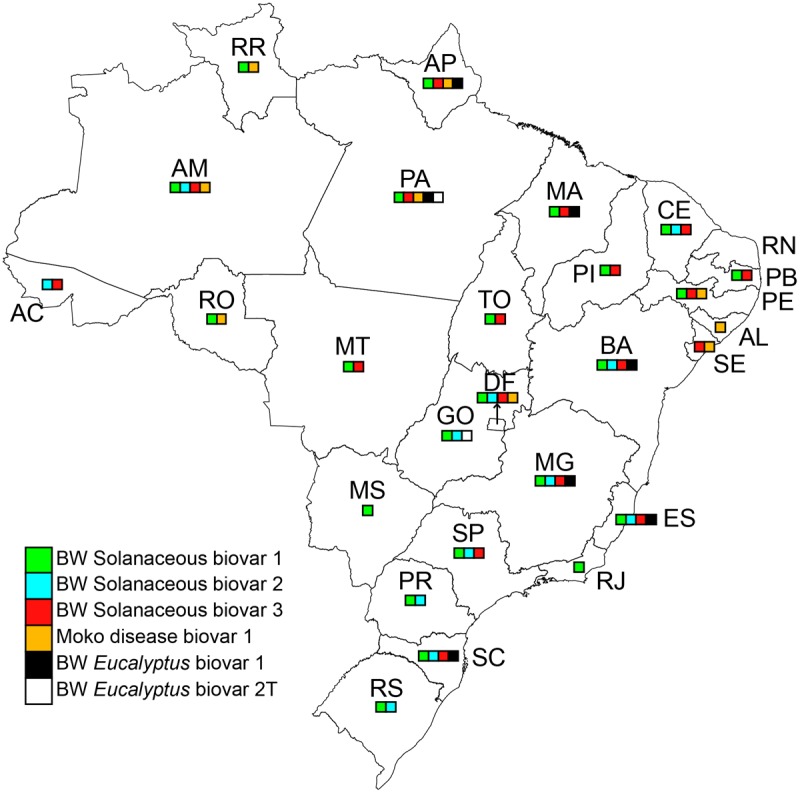
Brazilian map with state division representing the presence of *Ralstonia* species and diversity considering the reported data at the time and the list of isolates from the Embrapa Hortaliças collection, Albuquerque et al. (2014), Morais et al. (2015), and Santiago et al. (2017).

According to the Brazilian Ministry of Agriculture, a decade ago Moko was present in some of the northern and northeastern Brazilian states (MAPA, 2007). Later, in 2009, the disease was reported in two other states: in banana, in Alagoas (Andrade et al., 2009) and in two *Heliconia* species and in an ornamental *Musa* (*M. coccinea*) in the Federal District (Zoccoli et al., 2009; **Figure 1**).

Moko was first suspected to occur in Brazil in the 1930’s, when plants in the lowlands of the State of São Paulo displayed wilt symptoms (Wardlaw and Mcguire, 1933; Deslandes, 1938). The etiology of the disease, however, was controversial, since disease did not progress and could not be detected in extensive new surveys (Matallo and Martinez, 1978; Martinez, 1981). In 1972, suspected banana samples were collected in the irrigated perimeter in the states of Ceará and Paraíba (Ponte and Freire, 1972). Again, the diagnosis was not confirmed, and wilting was attributed to isolates of *R. solanacearum* other than race 2, the reason why epidemics probably did not progress (Matallo and Martinez, 1978; Martinez, 1981).

The first conclusive proof that Moko disease occurs in Brazil is credited to Tokeshi and Duarte (1976), who isolated the pathogen from a sample of cultivar Prata collected in the State of Amapá. The strain was identified as race 2 strain SFR (Robbs, 1981). This finding caused concern among banana growers in the State of São Paulo, who were regularly importing banana plants from farms located in the North Region. As a consequence, a working group of specialists was formed in 1977 to coordinate an extensive survey to assess the infestation of areas in the vicinities of the identified foci. This survey also included the border of Peru and Colombia, where technicians indicated that the disease was already spreading into the Brazilian side. Even though the disease was found in only one municipality on the border, the grower’s information was that a severe disease, called “banana fever,” had destroyed many plantations in the past, with symptoms that matched those of Moko (Mello, 1981). The main production region of Amazonas surveyed had close to 80% of the visited fields infested, some of them completely destroyed by Moko, which indicates that the disease could have been around well before its official report (Mello, 1981). The only state in the North Region where Moko has not been reported is Acre.

### *Eucalyptus* spp.

*Eucalyptus* sp. is among the few trees susceptible to infection by *Ralstonia* species. Already detected in several countries, BW was first reported in 1980 in Brazil by Sudo et al. (1983), a few years before the apparent first world record from the province of Guangdong-China (Wu and Liang, 1988). The disease was described in Prata, MG, in plants showing symptoms of wilting of the upper leaves, leaf yellowing, and scorching, as well as vascular discoloration. Another focus of the disease was detected in 1984 by Dianese and Takatsu (1985), in the region of Monte Dourado, State of Pará, in plants of *E. urophylla* and in a hybrid of *E. urophylla* ×*E. grandis*, in a plantation with approximately 30% of wilted plants.

In an attempt to understand the diversity of the *Ralstonia* complex infecting *Eucalyptus* species, Distrig et al. (1988) showed a variable virulence response of isolates in tomato, pepper and eggplant. All isolates were characterized as biovar 1, but with a different profile when studied by membrane protein polyacrylamide electrophoresis and compared to biovar 1 isolates from other hosts. Marques et al. (2012) reported the existence of biovar 2T isolates infecting *Eucalyptus*, and Marques et al. (2013) showed a higher virulence of biovar 2T isolates than those from biovar 1. Fonseca et al. (2014) showed a predominance of biovar 1 isolates, although molecular analysis indicated that most isolates had unknown sequevars, indicating that the diversity could be greater than that previously indicated based on race and biovar classifications.

After the first report in Minas Gerais, epidemics of eucalypt BW were observed in the States of Bahia (Robbs et al., 1988), Espírito Santo and Maranhão (Alfenas et al., 2006), Santa Catarina (Auer et al., 2008), and Amapá (Fonseca et al., 2014). Outbreaks in several infested eucalyptus nurseries in these states resulted in losses of approximately US$ 2.7 million (Alfenas et al., 2006), raising the importance of adopting strict control measures, such as preventing the bacterium from gaining access to production systems through infected seedlings, running water, and contaminated substrate. Unexpected and frequent BW outbreaks suggest that breeding resistant varieties might soon be an important aid for integrated disease management.

## Distribution of *Ralstonia* Species in Brazil

The distribution information is based on a literature review and on a data set of the bacterial collection of *Ralstonia* spp. isolates maintained at Embrapa Hortaliças, Brasília, DF. The collection contains 650 isolates obtained in 24 out of 26 Brazilian states. Twenty-two states harbored biovar 1 (phylotype II) in solanaceous plants; 12 states biovar 2 (phylotype II) in solanaceous plants; 17 states *R. pseudosolanacearum* (biovar 3, phylotype I) in solanaceous plants; 9 states Moko disease (race 2, biovar I, phylotype II); 7 states *R. solanacearum* biovar 1 (phylotype II) in *Eucalyptus*; and 2 states *R. solanacearum* biovar 2T (phylotype II) in *Eucalyptus* (**Figure 1**).

From the same collection, Santiago et al. (2017) and Albuquerque et al. (2014) identified the sequevars of 336 isolates of *Ralstonia* spp. Sixteen were identified: 1, 4, 6, 7, 18, 24, 25, 27, 28, 41, 50, 53, 54, 55, 56, and 57. As expected from the phylotype division by regions (Wicker et al., 2012), a large number of the sequevars are in phylotype II, originating in the Americas. For *R. pseudosolanacearum* (phylotype I), only sequevar 18 was reported, indicating no diversity associated with the probable recent entry of this phylotype in Brazil (Santiago et al., 2017).

## Current Situation of Main *Ralstonia* spp. Hosts in Brazil

**Tomato –** BW is one of the main diseases of tomato in the central and southern regions, except for processing tomatoes, which are usually planted after a grass crop in Central Brazil. It has become an increasing threat in protected crops where rotation is seldom used (Lopes et al., 2015). Control through grafting plants on top of BW-resistant rootstocks is only partial, since resistance can be overcome when the inoculum dose in the soil is high, the environmental conditions are favorable or the strain overcomes the resistance (Lopes and Mendonça, 2014; Lopes et al., 2015). The disease is a limiting factor for the humid and warm areas in the northern and northeastern regions, which depend on tomato imports from southern states (Lopes, 2015). Disease management through cultural practices is mandatory. Stable rootstock resistance derived from wild solanaceous species is a promising alternative.

**Potato –** In Brazil, the crop is traditionally grown in high latitudes in the southern and southeastern states. More recently, it has moved to the flat highlands of the central and northeastern states. In all these areas, phylotype II (biovars 1 and 2) prevails (Lopes, 2005). Serious BW outbreaks usually occur in ware potato crops during wet summers, in a combination of poor-quality seeds, insufficient crop rotations, and weather conditions favorable to disease onset and spread.

**Banana –** Moko disease is restricted to the North and Northeast Regions, where it has been kept under control through agronomical practices, especially avoiding the spread of diseased planting material (MAPA, 2007; Albuquerque et al., 2014). Efforts to develop resistant cultivars are scarce and not a priority in breeding programs, especially because good sources of resistance are not available. Studies on the variability of the pathogen, aiming to develop diagnostic kits for quick and reliable detection of diseased planting materials are important tools to prevent pathogen spread to non-infested areas (Pinheiro et al., 2011).

**Eucalyptus –** The disease is still restricted to some areas subject to warm and humid conditions, but outbreaks have been constantly rising in recent years. The main concern is to avoid the pathogen’s spread through stressed contaminated rooted stem cuttings in nurseries. Diagnostic and detection kits to index the planting stocks are required. Development of resistant cultivars is a viable long-term goal if sources of stable resistance are explored.

Since the first report of the disease, there has been a persistent search for resistant eucalyptus genotypes that might aid in reducing losses (Dianese et al., 1990; Fonseca et al., 2016). Resistance, along with preventive measures, may minimize the BW threat to this crop. Until now, only phylotype II was reported causing BW on eucalyptus in Brazil, while in other parts of the world there are reports of phylotype I (Carstensen et al., 2016). Therefore, breeding for resistant eucalyptus should consider phylotypes I and II.

## Future Trends

Considering the high plasticity of the members of the *Ralstonia* spp. and expected global warming, BW disease, which is favored by high temperatures, is likely to become an increasing threat to many hosts and non-hosts worldwide, especially in tropical and subtropical countries. In Brazil, the hot and humid climate in the North Region makes it extraordinarily vulnerable to BW. Therefore, upon the favorable combination of this environment with the complementing factors of the disease triangle, host and pathogen, BW might be a limiting factor for many plants, even for some considered non-hosts today. On the pathogen side, special concern is addressed to staple foods, such as cassava and sweet potatoes, known to be hosts of specific strains not yet present in Brazil.

## Author Contributions

CL conceived and organized the manuscript content and performed the historical review. MR gathered the information on the distribution of the pathogen and prepared the figure. CL and MR prepared and reviewed the manuscript.

## Conflict of Interest Statement

The authors declare that the research was conducted in the absence of any commercial or financial relationships that could be construed as a potential conflict of interest.
